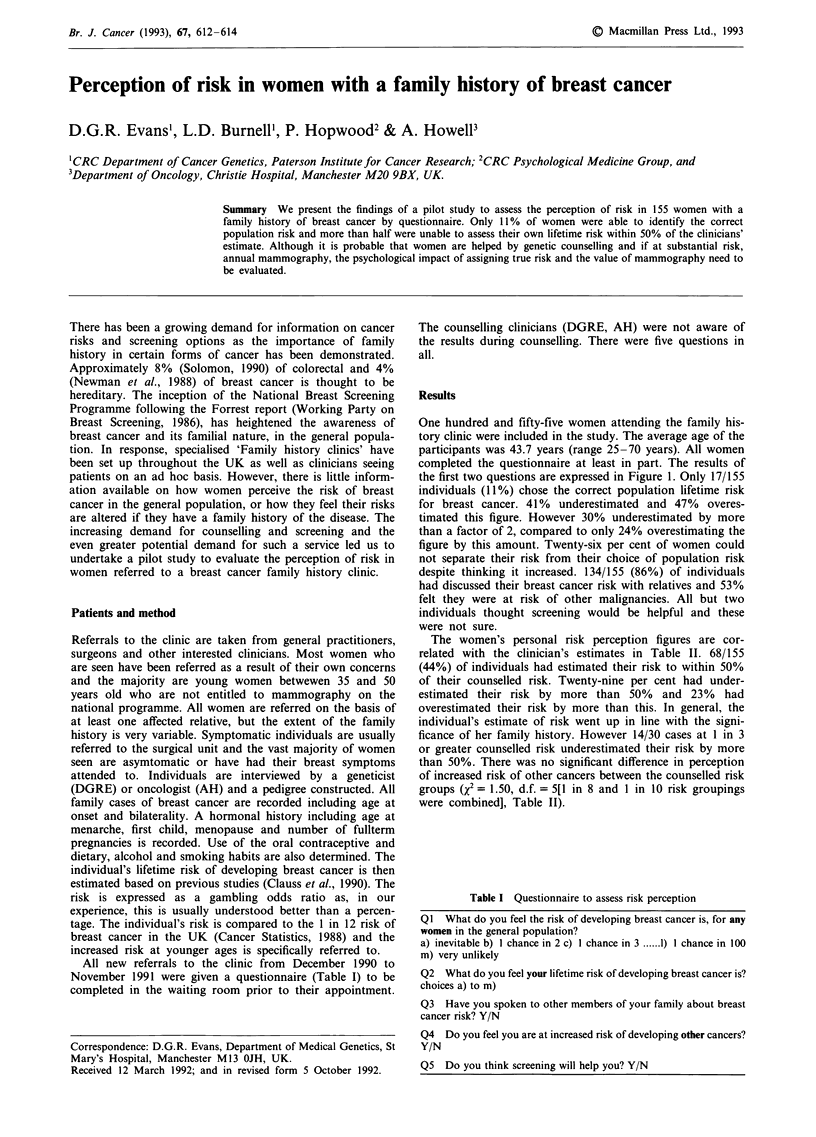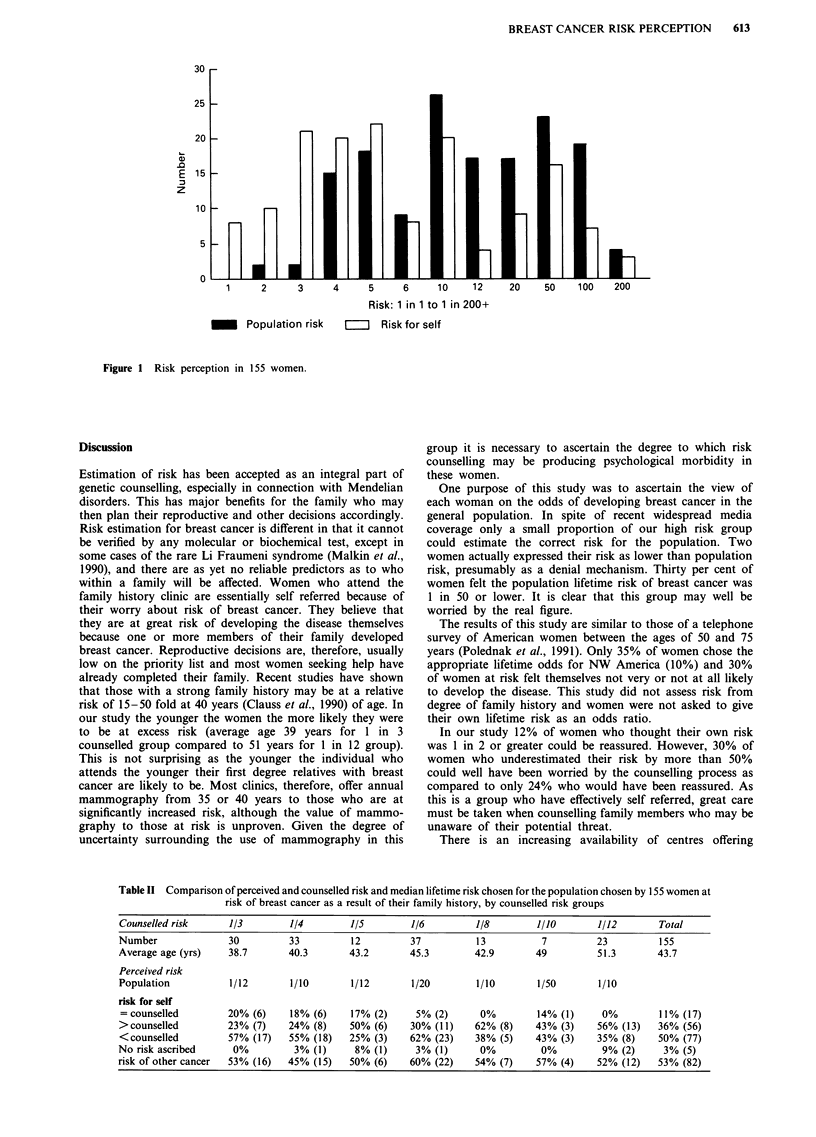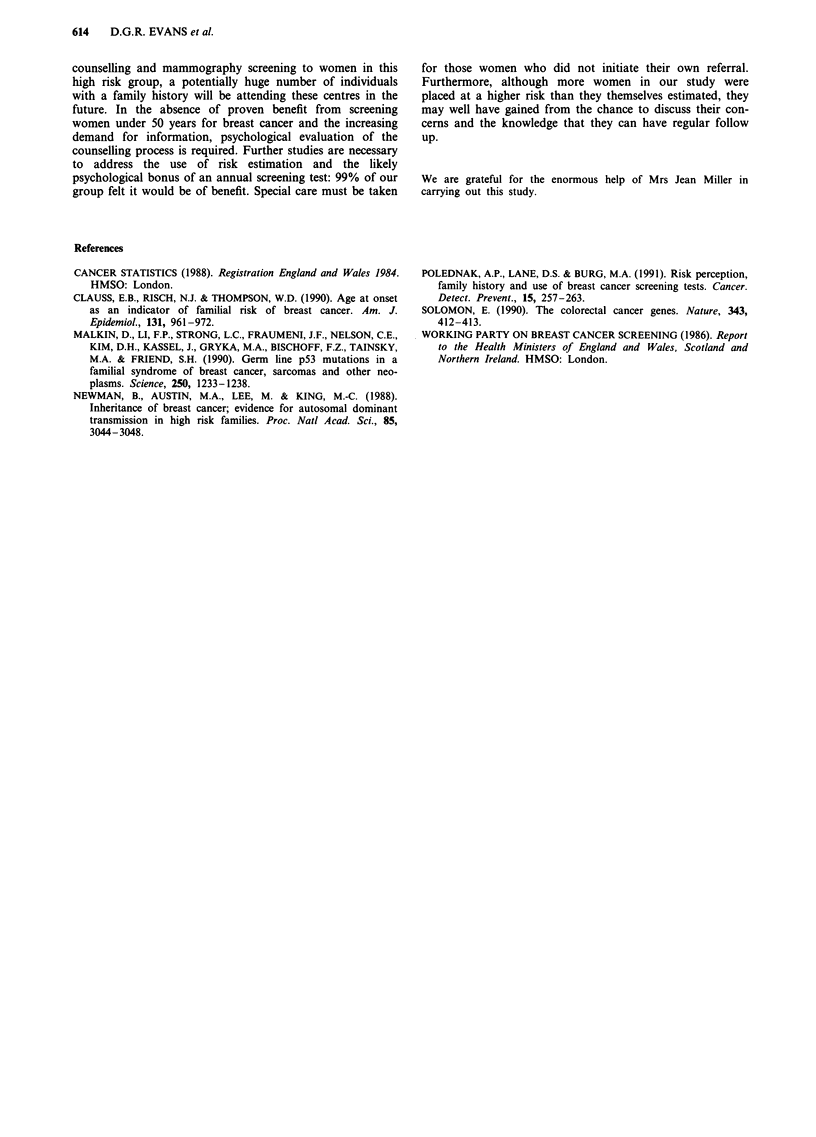# Perception of risk in women with a family history of breast cancer.

**DOI:** 10.1038/bjc.1993.112

**Published:** 1993-03

**Authors:** D. G. Evans, L. D. Burnell, P. Hopwood, A. Howell

**Affiliations:** CRC Department of Cancer Genetics, Paterson Institute for Cancer Research, Manchester, UK.

## Abstract

We present the findings of a pilot study to assess the perception of risk in 155 women with a family history of breast cancer by questionnaire. Only 11% of women were able to identify the correct population risk and more than half were unable to assess their own lifetime risk within 50% of the clinicians' estimate. Although it is probable that women are helped by genetic counselling and if at substantial risk, annual mammography, the psychological impact of assigning true risk and the value of mammography need to be evaluated.


Br. J. Cancer (1993), 67, 612-614 © Macmillan Press Ltd., 1993

### Perception of risk in women with a family history of breast cancer

D.G.R. Evans[1], L.D. Burnell[1], P. Hopwood[2] & A. Howell[3]

[1]*CRC Department of Cancer Genetics, Paterson Institute for Cancer Research;* [2]*CRC Psychological Medicine Group, and* [3]*Department of Oncology, Christie Hospital, Manchester M20 9BX, UK.*

**Summary** We present the findings of a pilot study to assess the perception of risk in 155 women with a family history of breast cancer by questionnaire. Only 11% of women were able to identify the correct population risk and more than half were unable to assess their own lifetime risk within 50% of the clinicians' estimate. Although it is probable that women are helped by genetic counselling and if at substantial risk, annual mammography, the psychological impact of assigning true risk and the value of mammography need to be evaluated.

There has been a growing demand for information on cancer risks and screening options as the importance of family history in certain forms of cancer has been demonstrated. Approximately 8% (Solomon, 1990) of colorectal and 4% (Newman *et al.*, 1988) of breast cancer is thought to be hereditary. The inception of the National Breast Screening Programme following the Forrest report (Working Party on Breast Screening, 1986), has heightened the awareness of breast cancer and its familial nature, in the general population. In response, specialised 'Family history clinics' have been set up throughout the UK as well as clinicians seeing patients on an ad hoc basis. However, there is little information available on how women perceive the risk of breast cancer in the general population, or how they feel their risks are altered if they have a family history of the disease. The increasing demand for counselling and screening and the even greater potential demand for such a service led us to undertake a pilot study to evaluate the perception of risk in women referred to a breast cancer family history clinic.

### Patients and method

Referrals to the clinic are taken from general practitioners, surgeons and other interested clinicians. Most women who are seen have been referred as a result of their own concerns and the majority are young women betwewen 35 and 50 years old who are not entitled to mammography on the national programme. All women are referred on the basis of at least one affected relative, but the extent of the family history is very variable. Symptomatic individuals are usually referred to the surgical unit and the vast majority of women seen are asymtomatic or have had their breast symptoms attended to. Individuals are interviewed by a geneticist (DGRE) or oncologist (AH) and a pedigree constructed. All family cases of breast cancer are recorded including age at onset and bilaterality. A hormonal history including age at menarche, first child, menopause and number of fullterm pregnancies is recorded. Use of the oral contraceptive and dietary, alcohol and smoking habits are also determined. The individual's lifetime risk of developing breast cancer is then estimated based on previous studies (Clauss *et al.*, 1990). The risk is expressed as a gambling odds ratio as, in our experience, this is usually understood better than a percentage. The individual's risk is compared to the 1 in 12 risk of breast cancer in the UK (Cancer Statistics, 1988) and the increased risk at younger ages is specifically referred to.

All new referrals to the clinic from December 1990 to November 1991 were given a questionnaire (Table I) to be completed in the waiting room prior to their appointment. The counselling clinicians (DGRE, AH) were not aware of the results during counselling. There were five questions in all.

Correspondence: D.G.R. Evans, Department of Medical Genetics, St Mary's Hospital, Manchester M13 0JH, UK.
Received 12 March 1992; and in revised form 5 October 1992.

### Results

One hundred and fifty-five women attending the family history clinic were included in the study. The average age of the participants was 43.7 years (range 25-70 years). All women completed the questionnaire at least in part. The results of the first two questions are expressed in Figure 1. Only 17/155 individuals (11%) chose the correct population lifetime risk for breast cancer. 41% underestimated and 47% overestimated this figure. However 30% underestimated by more than a factor of 2, compared to only 24% overestimating the figure by this amount. Twenty-six per cent of women could not separate their risk from their choice of population risk despite thinking it increased. 134/155 (86%) of individuals had discussed their breast cancer risk with relatives and 53% felt they were at risk of other malignancies. All but two individuals thought screening would be helpful and these were not sure.

The women's personal risk perception figures are correlated with the clinician's estimates in Table II. 68/155 (44%) of individuals had estimated their risk to within 50% of their counselled risk. Twenty-nine per cent had underestimated their risk by more than 50% and 23% had overestimated their risk by more than this. In general, the individual's estimate of risk went up in line with the significance of her family history. However 14/30 cases at 1 in 3 or greater counselled risk underestimated their risk by more than 50%. There was no significant difference in perception of increased risk of other cancers between the counselled risk groups ($\chi^2 = 1.50$, d.f. = 5[1 in 8 and 1 in 10 risk groupings were combined], Table II).

**Table I** Questionnaire to assess risk perception

Q1 What do you feel the risk of developing breast cancer is, for **any women** in the general population?
a) inevitable b) 1 chance in 2 c) 1 chance in 3 ......l) 1 chance in 100 m) very unlikely

Q2 What do you feel **your** lifetime risk of developing breast cancer is? choices a) to m)

Q3 Have you spoken to other members of your family about breast cancer risk? Y/N

Q4 Do you feel you are at increased risk of developing **other** cancers? Y/N

Q5 Do you think screening will help you? Y/N

**Figure 1** Risk perception in 155 women.

### Discussion

Estimation of risk has been accepted as an integral part of genetic counselling, especially in connection with Mendelian disorders. This has major benefits for the family who may then plan their reproductive and other decisions accordingly. Risk estimation for breast cancer is different in that it cannot be verified by any molecular or biochemical test, except in some cases of the rare Li Fraumeni syndrome (Malkin *et al.*, 1990), and there are as yet no reliable predictors as to who within a family will be affected. Women who attend the family history clinic are essentially self referred because of their worry about risk of breast cancer. They believe that they are at great risk of developing the disease themselves because one or more members of their family developed breast cancer. Reproductive decisions are, therefore, usually low on the priority list and most women seeking help have already completed their family. Recent studies have shown that those with a strong family history may be at a relative risk of 15–50 fold at 40 years (Clauss *et al.*, 1990) of age. In our study the younger the women the more likely they were to be at excess risk (average age 39 years for 1 in 3 counselled group compared to 51 years for 1 in 12 group). This is not surprising as the younger the individual who attends the younger their first degree relatives with breast cancer are likely to be. Most clinics, therefore, offer annual mammography from 35 or 40 years to those who are at significantly increased risk, although the value of mammography to those at risk is unproven. Given the degree of uncertainty surrounding the use of mammography in this group it is necessary to ascertain the degree to which risk counselling may be producing psychological morbidity in these women.

One purpose of this study was to ascertain the view of each woman on the odds of developing breast cancer in the general population. In spite of recent widespread media coverage only a small proportion of our high risk group could estimate the correct risk for the population. Two women actually expressed their risk as lower than population risk, presumably as a denial mechanism. Thirty per cent of women felt the population lifetime risk of breast cancer was 1 in 50 or lower. It is clear that this group may well be worried by the real figure.

The results of this study are similar to those of a telephone survey of American women between the ages of 50 and 75 years (Polednak *et al.*, 1991). Only 35% of women chose the appropriate lifetime odds for NW America (10%) and 30% of women at risk felt themselves not very or not at all likely to develop the disease. This study did not assess risk from degree of family history and women were not asked to give their own lifetime risk as an odds ratio.

In our study 12% of women who thought their own risk was 1 in 2 or greater could be reassured. However, 30% of women who underestimated their risk by more than 50% could well have been worried by the counselling process as compared to only 24% who would have been reassured. As this is a group who have effectively self referred, great care must be taken when counselling family members who may be unaware of their potential threat.

There is an increasing availability of centres offering

**Table II** Comparison of perceived and counselled risk and median lifetime risk chosen for the population chosen by 155 women at risk of breast cancer as a result of their family history, by counselled risk groups

| *Counselled risk* | *1/3* | *1/4* | *1/5* | *1/6* | *1/8* | *1/10* | *1/12* | *Total* |
|---|---|---|---|---|---|---|---|---|
| Number | 30 | 33 | 12 | 37 | 13 | 7 | 23 | 155 |
| Average age (yrs) | 38.7 | 40.3 | 43.2 | 45.3 | 42.9 | 49 | 51.3 | 43.7 |
| *Perceived risk* | | | | | | | | |
| Population | 1/12 | 1/10 | 1/12 | 1/20 | 1/10 | 1/50 | 1/10 | |
| **risk for self** | | | | | | | | |
| = counselled | 20% (6) | 18% (6) | 17% (2) | 5% (2) | 0% | 14% (1) | 0% | 11% (17) |
| > counselled | 23% (7) | 24% (8) | 50% (6) | 30% (11) | 62% (8) | 43% (3) | 56% (13) | 36% (56) |
| < counselled | 57% (17) | 55% (18) | 25% (3) | 62% (23) | 38% (5) | 43% (3) | 35% (8) | 50% (77) |
| No risk ascribed | 0% | 3% (1) | 8% (1) | 3% (1) | 0% | 0% | 9% (2) | 3% (5) |
| risk of other cancer | 53% (16) | 45% (15) | 50% (6) | 60% (22) | 54% (7) | 57% (4) | 52% (12) | 53% (82) |

counselling and mammography screening to women in this high risk group, a potentially huge number of individuals with a family history will be attending these centres in the future. In the absence of proven benefit from screening women under 50 years for breast cancer and the increasing demand for information, psychological evaluation of the counselling process is required. Further studies are necessary to address the use of risk estimation and the likely psychological bonus of an annual screening test: 99% of our group felt it would be of benefit. Special care must be taken for those women who did not initiate their own referral. Furthermore, although more women in our study were placed at a higher risk than they themselves estimated, they may well have gained from the chance to discuss their concerns and the knowledge that they can have regular follow up.

We are grateful for the enormous help of Mrs Jean Miller in carrying out this study.

### References

CANCER STATISTICS (1988). *Registration England and Wales 1984*. HMSO: London.

CLAUSS, E.B., RISCH, N.J. & THOMPSON, W.D. (1990). Age at onset as an indicator of familial risk of breast cancer. *Am. J. Epidemiol.*, **131,** 961–972.

MALKIN, D., LI, F.P., STRONG, L.C., FRAUMENI, J.F., NELSON, C.E., KIM, D.H., KASSEL, J., GRYKA, M.A., BISCHOFF, F.Z., TAINSKY, M.A. & FRIEND, S.H. (1990). Germ line p53 mutations in a familial syndrome of breast cancer, sarcomas and other neoplasms. *Science*, **250,** 1233–1238.

NEWMAN, B., AUSTIN, M.A., LEE, M. & KING, M.-C. (1988). Inheritance of breast cancer; evidence for autosomal dominant transmission in high risk families. *Proc. Natl Acad. Sci.*, **85,** 3044–3048.

POLEDNAK, A.P., LANE, D.S. & BURG, M.A. (1991). Risk perception, family history and use of breast cancer screening tests. *Cancer. Detect. Prevent.*, **15,** 257–263.

SOLOMON, E. (1990). The colorectal cancer genes. *Nature*, **343,** 412–413.

WORKING PARTY ON BREAST CANCER SCREENING (1986). *Report to the Health Ministers of England and Wales, Scotland and Northern Ireland*. HMSO: London.